# Ultrasound-Assisted Extraction, Characterization, and Antioxidant Activities of the Polysaccharides from Fermented *Astragalus membranaceus*

**DOI:** 10.3390/molecules30051159

**Published:** 2025-03-04

**Authors:** Jingyan Zhang, Zijing Liang, Kang Zhang, Xi Tang, Lei Wang, Xueyan Gu, Huub F. J. Savelkoul, Jianxi Li

**Affiliations:** 1 Engineering and Technology Research Center of Traditional Chinese Veterinary Medicine of Gansu Province, Lanzhou Institute of Husbandry and Pharmaceutical Sciences, Chinese Academy of Agricultural Sciences, Lanzhou 730050, China; zijingliang@126.com (Z.L.); 15719319192@163.com (K.Z.); syyxuan_040304@163.com (X.T.); wanglei807@126.com (L.W.); 158036854752@163.com (X.G.); 2 Cell Biology and Immunology Group, Wageningen University & Research, 6700 AH Wageningen, The Netherlands; huub.savelkoul@wur.nl

**Keywords:** ultrasound-assisted extraction, polysaccharides from fermented *Astragalus membranaceus*, *Astragalus* polysaccharides, characterization, antioxidant activities

## Abstract

This study aimed to optimize the ultrasound-assisted extraction (UAE) of polysaccharides from fermented *Astragalus membranaceus* (FAPS) and to investigate the physicochemical properties and antioxidant activities of the extracted polysaccharides. Using a combination of single-factor experiments and response surface methodology based on a Box–Behnken design, we improved the extraction of crude FAPS without deproteinization. Under optimal conditions (50 °C, 60 min, 8 mL/g, 480 W), the yield of crude FAPS obtained by UAE (7.35% ± 0.08) exceeded the yield from convectional hot water extraction (6.95% ± 0.24). After protein removal, the FAPS was subjected to comprehensive chemical analyses, including HPLC, HPGPC, FT-IR, UV spectroscopy, and a Congo red assay. The results showed that FAPS had a significantly higher carbohydrate content compared to the non-fermented group (95.38% ± 6.20% vs. 90.938% ± 3.80%), while the protein content was significantly lower than that of the non-fermented *Astragalus* polysaccharides (APS) group (1.26% ± 0.34% vs. 6.76% ± 0.87%). In addition, FAPS had a higher average molecular weight and a lower Mw/Mn ratio compared to APS. The primary monosaccharides in FAPS were identified as Glc, Ara, Gal and GalA, with a molar ratio of 379.72:13.26:7.75:6.78, and FAPS lacked a triple helix structure. In vitro, antioxidant assays showed that FAPS possessed superior antioxidant properties compared to APS. These results emphasize the significant potential of FAPS as an antioxidant, possibly superior to that of APS. The results of this study suggest that fermentation and UAE offer promising applications for the development and utilization of *Astragalus membranaceus* for human and animal health.

## 1. Introduction

*Astragalus membranaceus*, commonly known as *Radix Astragali* or Huang Qi in traditional Chinese medicine, is the root of a perennial flowering herb native to the northern and eastern regions of China, as well as Mongolia and Korea. It has been used as a medicine and food supplement for more than two thousand years [1]. It is often used to support immune function, reduce fatigue, and protect against stress. Additionally, some studies indicate that it may have cardioprotective effects, helping to improve heart function and lower blood pressure [2]. *A. membranaceus* contains various active compounds, including polysaccharides, saponins, flavonoids, and amino acids, which contribute to its therapeutic effects [3].

Astragalus polysaccharide (APS), as the key bioactive component extracted from *A. membranaceus,* has a wide range of health benefits, particularly in immune modulation, antioxidant activities, anti-inflammatory effects, antitumor properties, cardioprotective effects, antidiabetic properties, anti-aging effects and liver protection [1]. It is a high-molecular-weight compound composed of various sugar units, including glucose, galactose, arabinose, rhamnose, and xylose, and its structure is highly branched, which contributes to its biological activity [4]. The traditional method for extracting APS is hot water extraction. Nevertheless, this technique suffers from several drawbacks, such as low extraction efficiency, lengthy processing times, and the need for high temperatures [5]. Additionally, the prolonged extraction periods and excessive heat may result in the degradation of these polysaccharides, thereby diminishing their pharmacological activity [6]. Recent studies have predominantly concentrated on refining extraction techniques to enhance both the yield and bioactivity of APS. A variety of innovative extraction methodologies have been investigated and refined, including enzyme-assisted extraction, homogenization-assisted negative pressure cavitation extraction, and ultrasound assisted extraction (UAE), among others [3,7]. Among these methods, UAE offers several advantages over traditional extraction methods, including reduced extraction time, lower solvent consumption, and improved extraction efficiency [8]. Moreover, it is considered an environmentally friendly approach due to its minimal use of organic solvents and energy. It was reported that the extraction rate of APS using UAE was approximately 8% to 9%, and this yield was comparable to those obtained with enzyme assisted and alkaline alcohol extraction methods, and it is twice as effective as the hot water extraction followed by alcohol precipitation method [7]. But even so, the yield of APS needs to be improved and the purification process needs to be simplified.

Fermentation has proven to be a useful processing technology in the preparation of Chinese medicines of different functionalities [9]. First, it can promote the release of the active ingredients of Chinese herbs since microorganisms can produce some extracellular enzymes (such as protease, cellulase and amylase) that can digest the plant cell wall to promote the release of the relevant active ingredients [10]. In addition, fermentation can produce new active ingredients, like exopolysaccharides (EPS) produced by some bacterial species used in food applications, such as *streptococci* and *lactobacilli* [11]. EPS produced by lactic acid bacteria have garnered significant attention due to their diverse bioactivities (such as immunomodulatory effects, antioxidant activity, and antitumor properties), which have health benefits and potential therapeutic applications [12].

In previous studies, we developed a liquid fermentation-assisted extraction method to enhance the yield and bioactivity of polysaccharides derived from *A. membranaceus*, which proved to be both milder and more efficient compared to conventional techniques. The results revealed a 2.7-fold increase in the yield of polysaccharides from fermenting *A. membranaceus* (FAPS) with *Streptococcus alactolyticus* strain FGM [13]. Additionally, the extracted polysaccharides demonstrated a stronger promotive effect on the maturation of bone marrow-derived dendritic cells (BMDCs) compared to conventionally extracted APS [14]. Moreover, FAPS exhibited significant anti-hepatic fibrosis activity, potentially mediated through the elimination of oxygen free radicals in vivo [15]. Based on these findings, we are hoping to further optimize the extraction process of FAPS by integrating ultrasound-assisted extraction technology, thereby providing a more advanced and mature technique for future applications. However, there are no relevant studies that have been reported for fermentation-ultrasound assisted extraction technology applied to APS.

**Figure 1:**
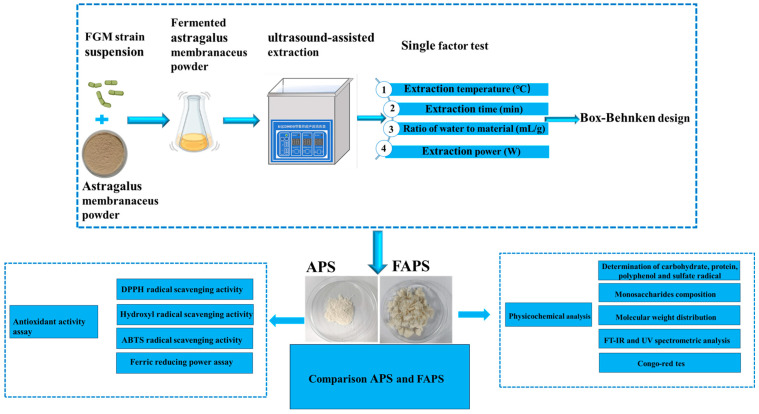
Flow chart of UAE and antioxidant activity analysis of FAPS.

The specific composition and structure of APS can vary depending on the extraction method [3], and the relationship between structure and activity needs to be further explored. Our investigation indicated that the increased FAPS was linked to a high metabolic flux toward exopolysaccharide (EPS) formation, which appeared to be regulated by the dexB and galE genes in strain FGM [16]. Consequently, we hypothesize that the composition, structure, and activity of FAPS may differ from those of APS extracted using conventional techniques. Therefore, this study was conducted in two sequential phases: initial systematic optimization of UAE parameters for crude FAPS, followed by a comprehensive comparative analysis of the chemical profiles, monosaccharide constituents, molecular weight distributions, and antioxidant capacities between conventional APS and FAPS, as schematically presented in Figure 1. The findings aimed to provide a robust data foundation for the efficient extraction and further development and utilization of fermented *Astragalus polysaccharides*.

## 2. Results and Discussion

### 2.1. Effects of Single-Factor Analysis on the Yield of Crude FAPS

**Figure 2:**
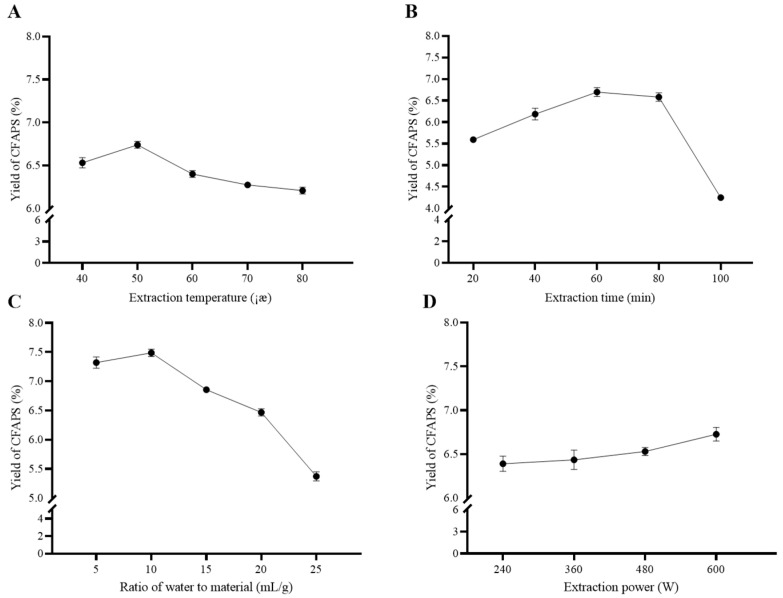
Effects of different extraction temperatures (**A**), extraction times (**B**), ratios of water to material (**C**) and extraction powers (**D**) on the yield of crude FAPS (CFAPS).

Temperature has been considered an important parameter in the extraction process of polysaccharides. To investigate the effect of temperature on the yield of crude FAPS, the extraction was carried out under different extraction temperatures (40, 50, 60, 70 and 80 °C). The other extraction parameters were set as follows: time was 60 min, the ratio of water to material was 15 mL/g and the power was 360 W. As shown in Figure 2A, the yield of polysaccharides increased initially with the increase of temperature from 40 to 50 °C, and then decreased with further increasing extraction temperatures. This trend showed that the temperature initially had a positive effect on the yield up to 50 °C because of the increase in the release rate, but later the yield was decreased, which was found to agree with previous reports [17]. The structure of the polysaccharides could be destroyed by excessively increased temperatures. Different polysaccharides exhibit varying sensitivities to temperature, and most polysaccharides undergo irreversible degradation once their tolerance temperature is exceeded. These degradation processes can affect the physicochemical properties of polysaccharides, such as solubility, viscosity, and bioactivity. For example, sodium alginate undergoes molecular chain breakage at high temperatures (above 80 °C), especially under acidic or alkaline conditions, where high temperatures accelerate its degradation process, and this degradation affects its gel-forming ability and viscosity [18]. Dextran undergoes thermal degradation at high temperatures (above 100 °C), leading to a reduction in its molecular weight, and this degradation can negatively impact its performance in drug delivery and biomedical applications [19]. We therefore set the temperature in the following experiments to 50 °C to compare the other parameters.

Figure 2B shows the impact of time, by using different extraction times (20, 40, 60, 80 and 100 min), on the yield of crude FAPS. The other extraction parameters were set as follows: the temperature was 50 °C, the ratio of water to material was 15 mL/g and the power was 360 W. The yield of crude FAPS increased with increasing extraction times from 20 to 60 min, but after that, there was an obvious decreasing tendency from 80 to 100 min. It has been reported that an appropriately prolonged extraction time could thereby enhance the diffusion of polysaccharides from the interior of plant cells [20]. However, prolonged ultrasonic exposure may result in the degradation of polysaccharide molecular structures, consequently diminishing extraction yields and potentially compromising the bioactivity of the polysaccharides. The findings in this study are consistent with previous studies, as outlined below. The recovery of APS from *A. membranaceus* var. *Mongholicus* increased with ultrasonic irradiation time, but after 50 min, the increasing trend was no longer significant and attenuated [21]. Chen et al. optimized the ultrasound-assisted extraction process for *Lycium barbarum* polysaccharides and found that excessively long ultrasonic durations (over 40 min) could lead to the breakage of polysaccharide molecular chains, thereby reducing both the extraction yield and the antioxidant activity of the polysaccharides [22]. Therefore, a time range of 40–80 min was implemented as optimal in the BBD experiments.

The other extraction parameters were set as follows. Fixing the temperature to 50 °C, time to 60 min and the power to 360 W allowed the study of the influence of water to material ratio (5, 10, 15, 20 and 25 mL/g) on the yield of crude FAPS, which is depicted in Figure 2C. The yield of crude FAPS increased slightly with the ratio of water to material from 5 to 10 mL/g, after which the yield decreased quickly. A larger water to material ratio indicates larger concentration differences between the interior of plant cells and the exterior solvent, and increased diffusion of polysaccharides, resulting in an enhanced yield of polysaccharides [23]. However, when the water to material ratio was increased further, the ultrasonic energy deposited in the volume would decrease, resulting in a decrease of extraction yield [24]. Hence, the range 5–15 mL/g of water to material ratio was implemented as being optimal in the BBD experiments.

To estimate the effect of the power of the ultrasonic equipment on the yield of crude FAPS, the extraction processes were carried out under different power settings (240, 360, 480 and 600 W), while the other extraction parameters were fixed as follows: temperature 50 °C, time 60 min and water to material ratio 15 mL/g. As illustrated in Figure 2D, the yield of crude FAPS exhibited a slight increase as the extraction power was elevated from 240 to 600 W. This observation suggests that higher extraction power positively influences the yield of crude FAPS, as enhanced ultrasonic power facilitates the rapid disruption of plant cells, thereby promoting the release of intracellular molecules into the solvent [25]. The maximum yield of crude FAPS was at 600 W under the device constraints. So, a power range of 360–600 W was implemented as being optimal in the BBD experiments.

### 2.2. Model Fitting and Statistical Analysis

According to the single factor results, while retaining the bioactivity of polysaccharides, the extraction temperature was set as 50 °C. The values of responses (yield of crude polysaccharides) at different experimental combinations are given in Table 1. The experimental results were fitted to the following second-order polynomial mode and analyzed by ANOVA in SPSS for the significance of the model (Equation (1)):

(1) $$\begin{array}{c} Y=7.52+0.12X_{1}-0.15X_{2}+0.11X_{3}-0.06X_{1}X_{2}-0.055X_{1}X_{3}-0.095X_{2}X_{3}\\ \;\;\;\;\;\;\;-0.41X_{1}^{2}-0.24X_{2}^{2}-0.22X_{3}^{2} \end{array}$$

where *Y* is the crude polysaccharides yield, X_1_ is the extraction time (min), X_2_ is the water to material ratio (mL/g) and X_3_ is the extraction power (W).

The BBD is commonly used to optimize extraction process variables, such as anthocyanins, polysaccharides, and phenolic compounds [26]. It is more effective than other methods and can be used to easily arrange and interpret results. The statistical significance of the model equation was evaluated by the F-test and the obtained *p*-value. In general, the model and corresponding variables would be more significant if the absolute F-value becomes greater and the *p*-value becomes smaller [27]. The high F-value (24.30) and small *p*-value (0.0002) in the results showed that the model was highly significant while comparing the relevant parameters (Table 2). The lack of fit indicates the failure of a model to represent data in the experimental domain at points not included in the regression. On the other hand, the small F-value (5.31) and high *p*-value (0.0703) indicated a lack of fit of the model, which was not significant. Furthermore, we found that the *p*-values of two independent variables (X_1_, X_2_) and three quadratic terms (X_1_^2^, X_2_^2^, X_3_^2^) were lower than 0.01 and the *p*-value of one linear term (X_3_) was lower than 0.05, indicating all three parameters have significant effects on the yield of crude FAPS. The determination coefficient (R^2^) and adjusted determination coefficient (Radj2) that reflect the proportion of variability in the data could be explained by the model [28]. In our study, the high value of R^2^ (0.9690) was close to 1.0, indicating a satisfactory correlation between the actual values and predicted values. The high value of Radj2 (0.9291) indicated that most of the variation in the yield of crude FAPS could be predicted by the model.

### 2.3. Analysis of Response Surface Plot and Contour Plot

**Figure 3:**
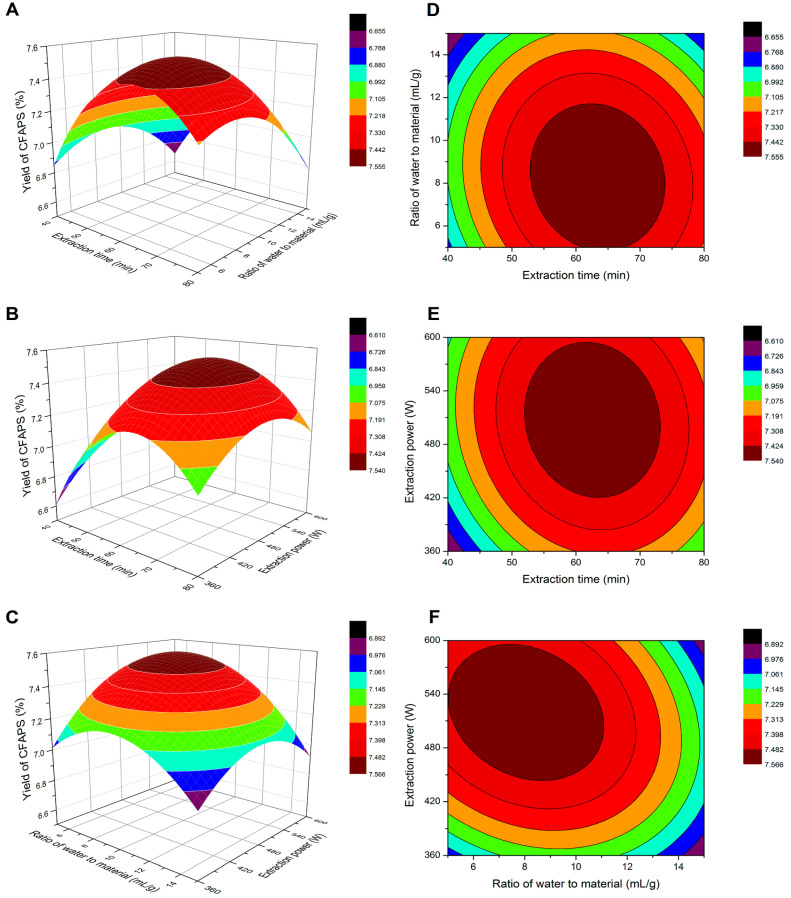
The three dimensional response surface plots and two dimensional contour plots show the interaction effects between water to material ratio and extraction time (**A**,**D**), extraction power and extraction time (**B**,**E**), extraction power and the ratio of material on the yield of CFAPS (**C**,**F**).

Three-dimensional response surface plots and two-dimensional contour plots are graphical representations of the regression equations, and they are thereby very useful to display the relationships between the independent variables and the response. Figure 3A,D show, the combined effect of extraction time (X_1_) and water-to-material ratio (X_2_) on the yield of crude FAPS, where the ultrasound extraction power (X_3_) was kept constant at the zero level. The response surface analysis revealed that the yield of crude FAPS initially increased with an increased extraction time in the range of 40–75 min, while the water-to-material ratio concurrently decreased within the range of 10–15 mL/g. Figure 3B,E shows the combined effects of the extraction time (X_1_) and the ultrasound extraction power (X_3_) on the yield of crude FAPS when the water to a material ratio (X_2_) was set at the level of zero. These images revealed that the yield of crude FAPS was increased gradually when the extraction power (X_3_) was increased from 360 to 480 W while the extraction time (X_1_) was prolonged from 40 to 70 min. Subsequently, the yield decreased gradually as the time increased further from 70 to 80 min. Figure 3C,F shows the combined effects of the ratio of water to material (X_2_) and the extraction power (X_3_) on the yield of crude FAPS, when the extraction time (X_1_) was fixed at the level of zero. The yield remained highest with the ratio within the range of 5–10 mL/g and the power within the range of 450–600 W.

Response surface methodology can be used as an effective, accurate, and simple tool for the evaluation of multiple parameters and their interactions [28]. The results in this section underline the importance of optimizing extraction parameters to achieve a balance between efficiency and polysaccharide integrity. More specifically, a longer extraction time initially increases the yield, but can lead to the degradation of the polysaccharide if extended beyond the optimal range (75 min). A lower ratio (5–10 mL/g) is favorable for maximizing the yield, as an excessive solvent volume dilutes the extract and reduces efficiency, and a higher power (450–600 W) improves the yield by enhancing cavitation effects, but excessive power or prolonged exposure may cause degradation of the polysaccharides.

### 2.4. Verification of the Predictive Model

Using the Design-Expert software, the optimum values of the extraction variables tested were determined for the crude FAPS with an extraction time of 60 min, a water-to-material ratio of 8 mL/g, and an ultrasound power of 519 W. Under these optimal conditions, the maximum yield of crude FAPS was 7.57%. To account for practical considerations, the optimal conditions were set to a temperature of 50 °C, an extraction time of 60 min, a water-to-material ratio of 8 mL/g, and an ultrasound power of 480 W. To validate the reliability and consistency of the model equation, three independent replicate experiments were conducted under these adjusted optimal conditions. The experimental yield of crude FAPS was determined to be 7.35% ± 0.08, demonstrating no statistically significant difference (*p* > 0.05) from the predicted yield. This strong correlation between experimental and predicted values confirms the validity and predictive accuracy of the developed extraction model. The comparative analysis revealed that the conventional hot water extraction method yielded 6.95% ± 0.24 of crude FAPS from fermented *Astragalus membranaceus*, while the optimized extraction protocol resulted in a significant improvement (*p* < 0.05) in extraction efficiency by about 5.8%.

### 2.5. Characterization of FAPS

As shown in Table 3, the total carbohydrate content (95.38% ± 6.20) and reducing sugar content (4.38% ± 0.08) of FAPS were significantly higher than those of APS (90.98% ± 3.80 and 1.96% ± 0.03, respectively). The protein content of FAPS (1.26% ± 0.34) was significantly lower than that of APS (6.76% ± 0.87). The content of sulfate radicals in FAPS (0.98% ± 0.06) was slightly lower than in APS (1.06% ± 0.04), but the difference was not statistically significant. Additionally, neither FAPS nor APS contained polyphenols. Studies have demonstrated that microbial fermentation can degrade glycoproteins and polysaccharide complexes in plant cell walls; thereby, proteins can more easily be removed through subsequent separation steps, such as precipitation, filtration, or chromatography. For instance, lactic acid bacteria fermentation has been shown to degrade proteins in soybean polysaccharides, resulting in increased polysaccharide purity (Reference: Food Chemistry, 2025 [11]). Furthermore, lactic acid bacteria fermentation can degrade proteins in plant polysaccharides, thereby improving both the purity and bioactivity of the polysaccharides (Reference: Journal of Agricultural and Food Chemistry, 2019 [29]). The potential mechanism may involve the secretion of various enzymes (such as proteases, glycosidases, and cellulases) during the fermentation process, leading to the breakdown of glycoprotein structures. Additionally, organic acids or other metabolic byproducts generated during fermentation may alter the pH or ionic strength of the system, further promoting the separation of proteins from polysaccharides. Combined with our previous study [30] we deduce that the lower protein concentration and higher total carbohydrate content in FAPS might be related to α-galactosidase and glucan-1, 6-α-glucosidase secreted by *Streptococcus alactolyticus* FGM strain.

**Figure 4:**
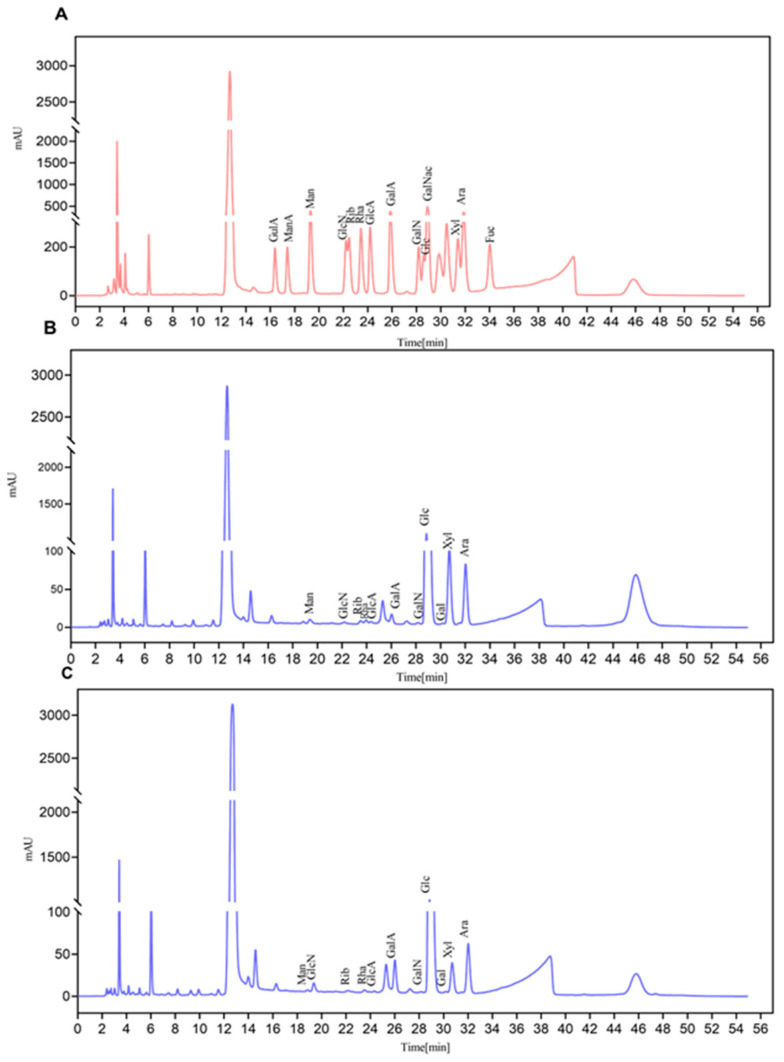
The HPLC chromatograms of monosaccharide standards (**A**), APS (**B**) and FAPS (**C**).

The HPLC chromatograms of monosaccharide standards, APS and FAPS are shown in Figure 4A–C. The monosaccharide composition of FAPS and APS was determined by a standard chromatogram in which the peak area reflecting predominantly the monosaccharide Glc, for both FAPS and APS, was similar to the result of purified polysaccharides from *A. membranaceus* by Yuge et al. [6]. As shown in Table 4, the main monosaccharide molar ratio of APS was Glc:Ara:Gal:GalA = 336.86:13.90:16.97:1.77, and the main monosaccharide molar ratio of FAPS was Glc:Ara:Gal:GalA = 379.72:13.26:7.75:6.78. This indicated that the most abundant sugar in APS was Glc, followed by Ara, Gal and GalA, which was consistent with the findings of Li et al. [31]. Compared with APS, the Glc content in FAPS was higher. This could be attributed to an ample amount of Glc as a carbon source in the initial conditions of the fermentation process, which was not fully utilized by the microorganisms. The Gal content in FAPS was lower than in APS, likely because Gal was catalyzed via the Leloir metabolic pathway, ultimately releasing ATP to power FGM [15]. It is also possible that during the fermentation process, Glc was converted to GalA under the action of galactose dehydrogenase. Moreover, GalA plays a role in antioxidant and antimicrobial activities [32].

**Figure 5:**
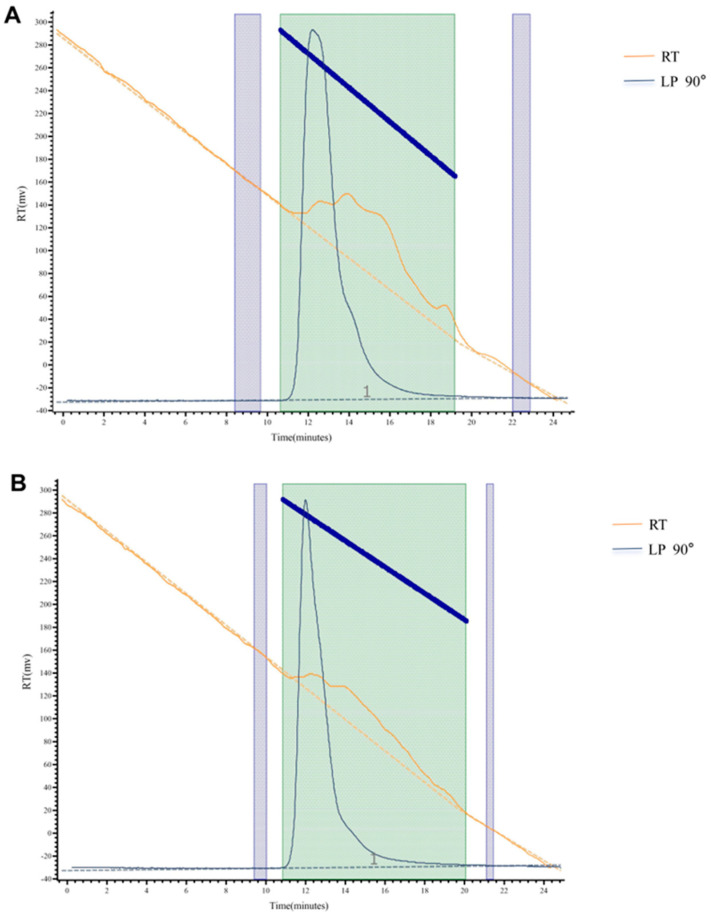
The HPGPC spectra of APS (**A**) and FAPS (**B**).

**Figure 6:**
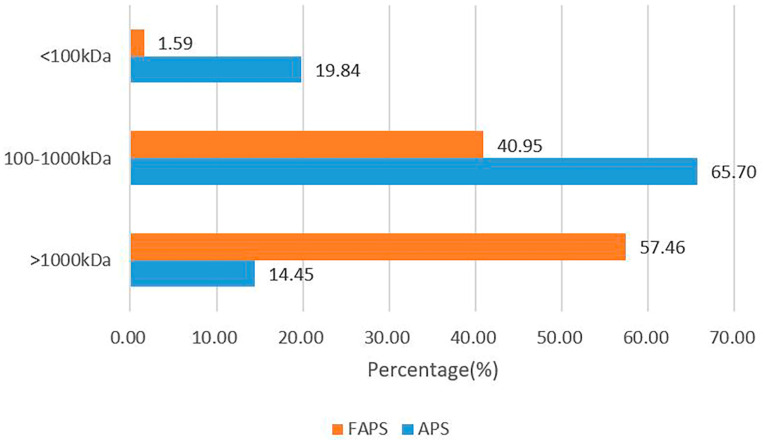
Molecular weight distribution of FAPS and APS.

As shown in Figure 5A,B, the HPGPC spectra of APS and FAPS each showed only one narrow peak, indicating that both APS and FAPS were homogeneous polysaccharides [33]. The difference in the Mw value of polysaccharides was related to the sugar composition, glycosidic bond and the connection mode of the branched chain [34]. A degree of dispersion (Mw/Mn) of polysaccharides is indicative of uniformity, and a degree of dispersion greater than unity reflects a wide molecular weight distribution [35]. It can be seen from Table 5 that the Mp, Mw and Mn of FAPS were higher than that of APS, but the PDI of FAPS was lower than that of APS, indicating better uniformity of FAPS. Using microorganisms for fermentation, the polysaccharide structure will be affected by the fermentation process [36]. It was reported that the Mn of EPS in lactic acid bacteria (LAB) was about 376–5210 KDa, and the Mw and PDI of APS were between 48–140 KDa and less than 23.0, respectively [37], which is similar to our findings [38]. As shown in Figure 6, the proportion of high molecular weight (>1000 kDa) components in FAPS was higher than in APS, and the proportion of medium molecular weight (100–1000 kDa) and lower molecular weight (<100 kDa) components in FAPS was lower than in APS. This is consistent with the data from Table 5. Therefore, it can be concluded that the fermentation of *A. membranaceus* with LAB strains increases the molecular weight of polysaccharides in the product.

**Figure 7:**
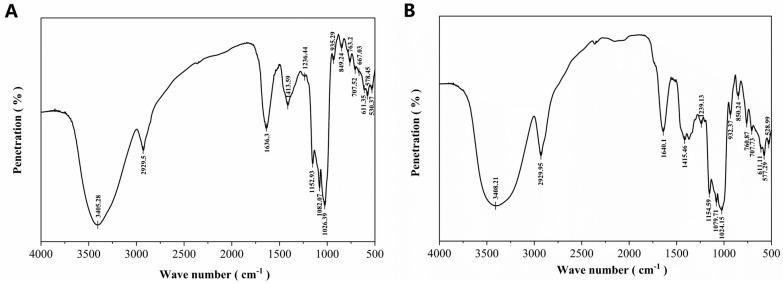
FT-IR spectrum of APS (**A**) and FAPS (**B**).

As shown in Figure 7, FAPS and APS had broad absorption peaks around 3405 cm⁻^1^, indicative of γ-OH stretching vibrations [39]. Both polysaccharides showed weak peaks around 2927 cm⁻¹, corresponding to γ-CH₂ and C-H stretching vibrations. The absorption peaks around 1640 cm⁻^1^ were attributed to the C-O stretching vibration in APS and FAPS [40]. The absorption peaks at 1413.31 cm⁻^1^ in APS and 1338.46 cm⁻^1^ in FAPS were attributed to δ-CH₂ bending vibrations [41]. The absorption peaks around 1024 cm⁻¹ in both FAPS and APS were associated with pyranose ring structures. Moreover, FAPS and APS both contain uronic acids, consistent with the monosaccharide composition data presented in Table 4. Additionally, the absorption peak at 1236.53 cm⁻^1^ indicated γ-C-O-C bending vibrations and the absorption peaks at 936.06 cm⁻^1^ and 846.04 cm⁻^1^ indicated the presence of α-configurations in APS. Moreover, FAPS exhibited an absorption peak at 1413.72 cm⁻^1^ due to carboxyl C-O vibrations. The peaks at 933.01 cm⁻^1^ and 849.44 cm⁻¹ suggested the presence of both α-D-pyranose and β-D-pyranose anomeric forms in FAPS [42].

**Figure 8:**
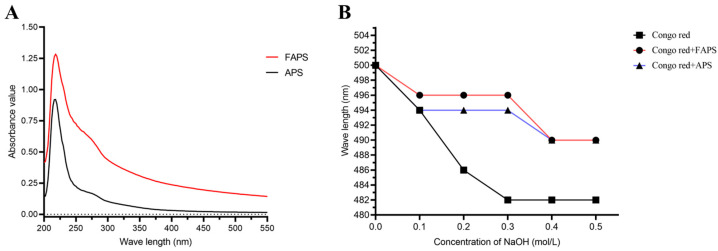
UV spectrum of FAPS and APS (**A**). Changes in absorption wavelength maximum of mixture of Congo red, FAPS and APS at various concentrations of NaOH (**B**).

As shown in Figure 8, APS and FAPS had no absorption peak at the wavelength of 280 nm, which is inconsistent with the chemical composition results in Table 3. Considering the removal of free proteins by the Sevage reagent and the protein content detected by the BCA method, it was speculated that APS might be a polysaccharide containing binding proteins, that is, a proteoglycan. The APS-gonorrhoin reagent and FAPs-gonorrhoin reagent did not form a complexation reaction that increased the maximum absorption wavelength, and the wavelength gradually decreased with the increase of the concentration of sodium hydroxide, indicating that neither of them contained a three-strand helical structure.

### 2.6. In Vitro Antioxidant Activity of FAPS

**Figure 9:**
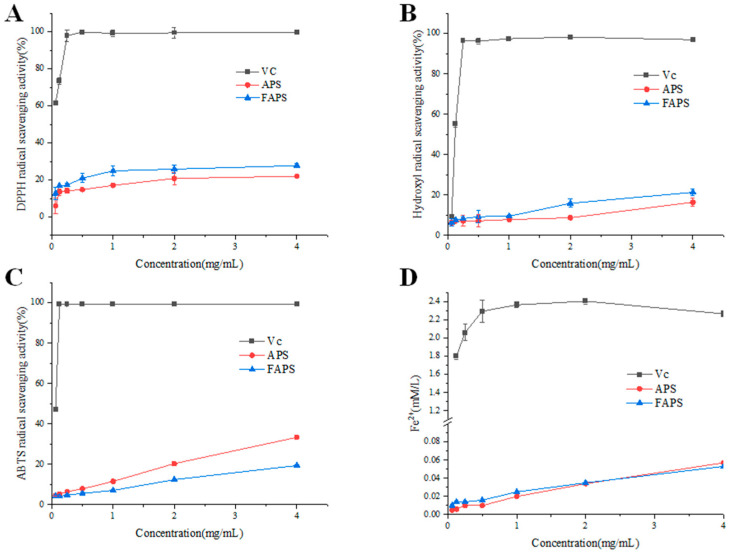
Scavenging effects of APS and FAPS at different concentrations on DPPH radical scavenging assay (**A**), hydroxyl radical scavenging assay (**B**), ABTS radical scavenging assay (**C**) and ferric reducing power assay (**D**).

DPPH is often employed as a representative reagent to examine the free radical scavenging activities of bioactive compounds. The antioxidant activity of polysaccharides is attributed to their hydrogen-donating capacity [43]. The DPPH radical is a stable proton radical with a maximum absorption peak at the wavelength of 517 nm. In an ethanol solution it appears purple, while the color will fade when a proton-donating substance is present. This change is very rapid and is highly sensitive for the detection of antioxidant activity [44]. As shown in Figure 9A, the DPPH free radical scavenging rates of APS and FAPS were increased in a quadratic concentration-dependent manner. The positive control Vc had a strong antioxidant activity (99.49%) at a concentration in solution of 4 mg/mL. However, the DPPH radical scavenging rates of FAPS and APS were 26.40% and 20.81%, respectively. It is reported that the carboxyl and hydroxyl groups in uronic acid provide more active sites for reacting with DPPH radicals [45]. Moreover, reducing sugars (such as glucose, fructose, etc.) can significantly enhance the scavenging ability of DPPH radicals by providing hydrogen atoms or electrons. For example, one study revealed that the antioxidant capacity of honey is positively correlated with its reducing sugar content, and the reducing sugars significantly enhanced the scavenging effect on DPPH radicals by providing hydrogen atoms or electrons [46]. Other studies evaluated the antioxidant activity of reducing sugars (such as glucose and fructose) and explored their roles in food systems, and found that reducing sugars effectively scavenged free radicals, and their activity exhibited a positive correlation with concentration [47,48]. The DPPH radical scavenging rate of FAPS was greater than APS, and the reason might be related to the increased GalA content and higher concentration of reducing sugars in FAPS. Moreover, proteins or polypeptides in FAPS also appear to increase the DPPH free radical detection of those macromolecules, thereby revealing their antioxidant activity [49].

Hydroxyl is the most active free radical, which reacts with various biological macromolecules in the body and can cause severe molecular damage [50]. The hydroxyl free radical scavenging capacity of Vc, FAPS and APS were all dose-dependent (Figure 9B), while the scavenging activity of FAPS was stronger than that of APS at the same concentration, and this was in concordance with the results of the DPPH radical scavenging assay. At a concentration of 4 mg/mL, the hydroxyl radical scavenging rate of FAPS (21.50%) was shown to be stronger than that of APS (16.49%), but it was far lower than that of Vc (96.91%). The hydroxyl radicals scavenging ability of these samples has a positive correlation with the increase in the number of uronic acid residues [28]. Uronic acid can reduce the generation of hydroxyl radicals either by directly scavenging them or by chelating metal ions. Additionally, uronic acid can activate the hydrogen atom at the anomeric carbon, and the activated hydrogen can mitigate oxidative damage either by directly reacting with hydroxyl radicals or by interrupting the free radical chain reaction [51]. Therefore, FAPS has relatively better hydroxyl radical scavenging activity and this may be due to its higher content of uronic acid as shown in Table 4. ABTS is a free and stable radical cation, and its oxidation produces a stable blue–green ABTS radical, with a maximum absorption peak at a wavelength of 734 nm. The staining of the solution fades and the absorbance decreases when an antioxidant is encountered and it can be used as an index reflecting the antioxidant activity [52]. As depicted in Figure 9C, the ABTS free radical scavenging capacity of Vc, APS and FAPS increased with increasing concentrations. At a concentration of 4 mg/mL, the ABTS radical scavenging rates of Vc, APS and FAPS were 99.39%, 33.38% and 19.43%, respectively. Hydroxyl groups play an important role in scavenging the ABTS free radicals [53]. FAPS showed no stronger ABTS radical scavenging ability than APS, and the reason may be that FAPS has relatively few hydroxyl groups to exhibit ABTS scavenging free radical activity.

The ferric-reducing power assay serves as a useful tool to detect antioxidant activity. The reducing power of polysaccharides is based on antioxidants that reduce Fe^3+^ in potassium ferricyanide to its ferrous form (Fe^2+^), and upon the addition of ferric chloride to further form Prussian blue, which has a maximum absorption peak at 700 nm. Thus, the reducing power of polysaccharides can be reflected by the magnitude of the absorbance [54]. As shown in Figure 9D, the reducing power of all the samples was increased in a dose-dependent manner. At a concentration of 4 mg/mL, the absorbance of Vc, FAPS and APS were 2.264, 0.053 and 0.057, respectively. Thus, it can be concluded that FAPS has no significant activity on ferric-reducing, similar to APS.

## 3. Conclusions

In the present study, the ultrasonic-assisted extraction parameters for the crude polysaccharides from fermented *A. membranaceus* with *Streptococcus alactolyticus* strain FGM were optimized by RSM with a BBD design, and the parameters were 50 °C, 60 min, 8 mL/g, 480 W. In contrast with the traditional hot water extraction, it not only saves significant time and energy but also shows a positive effect on the yield of crude FAPS. Regarding the physicochemical characterization of FAPS and conventional APS, there is differences in protein content and monosaccharide composition, two polysaccharide fractions didn’t exhibited structural characterization of polyphenolic compounds and three-strand helical structure. Moreover, FAPS has a higher content of total carbohydrate, GalA and Glc; and a lower proportion of medium-to-low molecular weight components. Both APS and FAPS have typical chemical structure characteristics of polysaccharides. FAPS showed better scavenging activity of DPPH radicals and hydroxyl radicals than APS, which is related to the higher uronic acid content in FAPS. Future studies will focus on further exploring the structural and functional properties of FAPS under these optimized conditions to ensure both high yield and bioactivity. Additionally, scaling up the extraction process while maintaining these optimal parameters should be investigated for potential industrial applications. These findings provide valuable insights into the beneficial applications of fermentation and UAE technologies in the product development of *A. membranaceus* and its polysaccharides, as well as for developing efficient and scalable extraction processes for bioactive polysaccharides.

## 4. Materials and Methods

### 4.1. Materials and Chemicals

*A. membranaceus* was purchased from the Yellow River medicinal material market of Lanzhou city in Gansu Province, China, and identified by Prof. Jifang Zheng, Lanzhou Institute of Husbandry and Pharmaceutical Sciences of Chinese Academy of Agricultural Sciences. *A. membranaceus* was dried for 24 h at 60 °C. Other materials and chemicals used in the present study were as follows: *S. alactolyticus* strain FGM (GenBank accession No. JX435470, China Patent No.20120141827.5) was isolated from chicken cecum and preserved at the Lanzhou Institute of Husbandry and Pharmaceutical Sciences of Chinese Academy of Agricultural Sciences. Dialysis tubing (molecular weight cut off, 7 KDa), vitamin C, 1,1-diphenyl-2-picrylhydrazy (DPPH), a BCA protein concentration determination kit (Biosharp Co., Ltd., China, Beijing), and 2,2-azino-bis-3- ethylbenzothiazoline-6-sulfonic acid (ABTS) were purchased from Solarbio Co., Ltd. (Beijing, China). Mannose (Man), ribose (Rib), rhamnose (Rha), glucuronic acid (GlcA), glucosamine (GlcN), galacturonic acid (GalA), galactosamine (GalN), glucose (Glc), galactose (Gal), xylose (Xyl), and arabinose (Ara) were purchased from Yuanye Biotechnology (Shanghai, China). All of the other chemicals and reagents were analytical grade.

### 4.2. Bacteria Cultures and Growth Condition

The cryopreserved FGM strain was resuscitated by inoculating 1 mL of bacteria solution into 50 mL MRS broth medium and anaerobically incubated at 37 °C for 24 h. Then, 1 mL of the resuscitated bacteria fluid was inoculated into 50 mL of MRS broth medium again, which represented the first generation, and it was anaerobically cultured at 37 °C for 12 h, and thereafter repeatedly passaged to the fourth generation (the bacterial count was about 4.5 × 10^8^ CFU/mL).

### 4.3. Preparation of Fermented Astragalus Membranaceus

Dried *Astragalus membranaceus* were ground and subsequently sieved through a 65 mesh screen (250 μm ± 9.9). The fermentation medium (China Patent No.201210141832.6) was placed in a fermenter (BIOF-6010B/G/A, Shanghai Gaoji Biological Engineering Co., Ltd., China, Shanghai) with *A. membranaceus* powder solution (8%, *w*/*v*) and autoclaved at 121 °C for 5 min. After the temperature dropped to 37 °C, the fourth generation (5%, *v*/*v*) of the FGM strain suspension was added and then adjusted to pH 7.4 and fermented at 37 °C, 200 rpm for 48 h. After fermentation, the fermentation products were subjected to vacuum freeze-drying using an LGJ-10F freeze dryer (Beijing Songyuan Huaxing Technology Development Co., Ltd., China, Beijing). The resulting product, namely fermented Astragalus powder, was stored at −20 °C for future use.

### 4.4. Preparation of FAPS and APS

The fermented Astragalus powder (50 g) was extracted twice with water (500 mL) in a water bath at 80 °C. The extracted liquids were combined and concentrated to 250 mL using a rotary evaporator at 60 °C under vacuum. The proteins in the extract were removed by Sevage reagent [17]. The protein was removed by adding 83.33 mL Sevage (chloroform:n-butanol 4:1) to the concentrated liquid using a separation funnel. The supernatant was collected and this procedure was repeated 6–8 times. The protein-removed solution was then placed in a dialysis tube with a molecular weight cut-off of 7 KDa, dialyzed for 48 h in running water, and then dialyzed against distilled water for 24 h. Subsequently, the polysaccharide was precipitated overnight at 4 °C after adding three times the volume of anhydrous ethanol. The precipitated polysaccharides were washed twice with anhydrous ethanol, twice with acetone and once with ether. After the ether volatilized completely, it was rinsed with distilled water and then freeze-dried to obtain FAPS. The preparation method of APS powder is consistent with the above procedures.

### 4.5. Experimental Design

#### 4.5.1. Ultrasonic Assisted Extraction

Polysaccharides were extracted with distilled water in an ultrasonic cleaner (KQ-600DE, Kunshan Ultrasonic Instruments Co., Ltd., China, Kunshan) whose temperature was preset before the extraction process. When the temperature reached the set value, the samples in the triangular flask were placed in the ultrasonic cleaner and a preset program was applied. After extraction, the extracts were filtered through gauze, centrifuged for 15 min at 4000 rpm and then concentrated to a quarter of the original volume by rotary evaporators. We did not carry out the deproteinization steps in the extracting process during the optimization of the method to reduce potential errors. The method of the remaining steps was the same as the hot water extraction, and we finally obtained crude FAPS. The polysaccharides yield (%) was calculated using the following equation (Equation (2)):

(2) $$Y\left(\%\right)=\frac{W_{1}\left(g\right)}{W_{2}\left(g\right)}\times 100$$

where *Y* is the yield of polysaccharides (%); *W*_1_ is the weight of extracted polysaccharides (g); and *W*_2_ is the weight of the dried *A. membranaceus* powder (g).

#### 4.5.2. Single Factor Experiment

The extraction temperature (40, 50, 60, 70, 80 °C), extraction time (20, 40, 60, 80, 100 min), ratio of water to material (5, 10, 15, 20, 25 mL/g) and extraction power (240, 360, 480, 600 g of weight W) can influence the yield of crude FAPS, so the effect of these four factors on extraction were studied by a single factor design: one factor was changed while the other factors were held constant in each experiment.

#### 4.5.3. Experimental Design of RSM

According to the results of the single-factor experiments, the optimal temperature was set as 50 °C, and a three-factors-three-levels Box–Behnken design (Design Expert Software, Version 8.0.5, Stat-Ease Inc., Minneapolis, MN, USA) was applied to determine the optimal ranges of the variables that significantly affected the extraction efficiency: time (X_1_, min), ratio of water to material (X_2_, mL/g) and power (X_3_, W). The design consisted of 17 experimental points in random order as shown in Table 1.

The experimental data were fitted to the following second-order polynomial mode (Equation (3)):

(3) $$Y=\beta_{0}+\sum_{i=1}^{3}\beta_{i}X_{i}+\sum_{i=1}^{3}\beta iiXi^{2}+\sum_{i=1}^{3}\sum_{j=i+1}^{3}\beta ijXiXj$$

where *Y* is the predicted response (experimental values). *β*_0_*, β_i_, β_ii_* and *β_ij_* are the model intercept coefficient, linearity, linear, quadratic and interactive coefficients, respectively; *X_i_* and *X_j_* are the coded independent variables; and the terms *X_i_^2^* and *X_i_X_j_* represent the quadratic and interaction terms, respectively.

### 4.6. Physicochemical Analysis

#### 4.6.1. Determination of Carbohydrate, Protein, Polyphenol and Sulfate Radical

The concentration of carbohydrates was determined as described before [55] with some modifications. The solution of polysaccharides (2 mL, 0.04 mg/mL) was mixed with 1 mL phenol solution (0.5%) and 5 mL sulfuric acid, and the reaction mixture was incubated in a boiling water bath for 15 min, after which the absorbance at the wavelength of 490 nm was recorded using a spectrophotometer. The amount of carbohydrates was determined from a standard curve generated using glucose as reference sugar. The concentration of protein was determined by a BCA protein concentration determination kit using bovine serum albumin as the standard. The concentration of polyphenol was determined by the method of Folin–Ciocalteu [30]. The concentration of sulfate radicals was determined according to the BaCl_2_–gelatin method as described previously [56].

#### 4.6.2. Monosaccharides Composition

The monosaccharides composition of FAPS and APS was analyzed by an HPLC method [57] with slight modifications. The Agilent 1200 Series HPLC system was equipped with an ultraviolet detector and a SHISEIDO-C18 (4.6 mm × 250 mm, 5 μm) capillary column. The mobile phase was a mixture of 0.1 M phosphate buffer (pH 6.9), 82% (solution A) and 18% acetonitrile (solution B). The flow rate was set at 1.0 mL/min and the wavelength of the ultraviolet detector was 245 nm. The FAPS powder (10 mg) was completely hydrolyzed to monosaccharides with 0.5 mL of trifluoroacetic acid (TFA, 4 M) at 121 °C for 2 h. The dried hydrolyzed polysaccharides sample and 10 standard monosaccharides were dissolved in 0.5 mL of NaOH (0.3 M) and 0.5 mL of 3-methyl-1-phenyl-5-pyrazolinone (PMP) solution (0.5 M with methanol as the solvent) and derivatized for 60 min at 70 °C. After cooling to room temperature, the mixtures were added to 0.5 mL of HCl solution (0.3 M) to stop the reaction, followed by extraction with 0.5 mL of chloroform three times. The aqueous layer was filtered through a 0.22 μm membrane and 10 μL of the filtrate was subjected to HPLC analysis. A mixture of each standard monosaccharide (mannose, ribose, rhamnose, glucuronic acid, galacturonic acid, glucose, galactose, xylose, arabinose and fucose) was subjected to the same condition as a reference. Measurement data are presented as molar ratios.

#### 4.6.3. Molecular Weight Distribution

The molecular weight distribution of FAPS and APS was determined by the HPGPC method [57] with slight modifications. An Aligent 1260 Infinity II MDS gel permeation chromatograph equipped with a differential detector, a dual-angle laser scattering detector, and a PL aquagel-OH Mixed-H column (7.5 × 300 mm, 8 μm, molecular weight range 500–10,000,000) was used for detection. The sample was dissolved in the mobile phase (deionized water with 0.01% sodium azide) to prepare a 1–3 mg/mL solution, and 20 μL was injected into the machine after passing through a 0.22 μm microporous membrane, while the column temperature was 45 °C and the flow rate was 1.0 mL/min.

#### 4.6.4. FT-IR and UV Spectrometric Analysis

According to the KBr disc method [57], 1 mg dried FAPS or APS was ground with 100 mg of KBr powder and pressed into tablets. Samples were measured using a Fourier transform infrared (FT-IR) spectrometer Nicolet Is5 (Thermo Fisher, Waltham, MA, USA) with a scanning range from 4000 to 400 cm^−1^. The ultraviolet–visible (UV-Vis) absorption spectra of the polysaccharide solutions (1 mg/mL) were recorded using a UV-Vis spectrophotometer (BioTek multi-mode reader) over the wavelength range of 200–550 nm. The Congo red test was used to measure the conformational structures of APS and FAPS with minor modifications [58] as follows: 2 mL polysaccharides solution (2.5 mg/mL) was mixed with 2.0 mL Congo red solution (100 μmol/L). Then, a NaOH solution (1 mol/L) was added gradually to the mixture. The final concentrations of NaOH were 0, 0.1, 0.2, 0.3, 0.4 and 0.5 mol/L, respectively. After keeping the mixture at room temperature for 5 min, the maximum absorption wavelength was identified in the wavelength range of 200–800 nm. The mixture without polysaccharides was used as a blank control.

### 4.7. Assay of Antioxidant Activity

#### 4.7.1. DPPH Radical Scavenging Activity

The DPPH radical scavenging activities of the polysaccharides were measured according to a previously reported method, with minor modifications [59] as follows: 20 μL of 0.4 mM DPPH ethanol solution and 100 μL of deionized water were added to 50 μL of sample water solution at various concentrations (0.0625, 0.125, 0.25, 0.5, 1, 2, 4 mg/mL). The mixture was shaken thoroughly and then incubated at room temperature for 30 min in the dark. The absorbance was measured at the wavelength of 517 nm. Vitamin C was used as a positive control. The DPPH free radical scavenging percentage was calculated by the following equation (Equation (4)):

(4) $$Scavenging\;activity\;(\%)=\frac{A_{0}-\left(A_{a}-A_{b}\right)}{A_{0}}\times 100$$

where *A*_0_ is the absorbance of the blank control (deionized water instead of sample solution), *A_a_* is the absorbance of the sample solution and *A_b_* is the absorbance of the sample under identical conditions as *A_a_* with ethanol instead of the DPPH solution.

#### 4.7.2. Hydroxyl Radical Scavenging Activity

The hydroxyl radical scavenging activity of FAPS and APS was evaluated according to the method of Gao et al. [60] with some changes. First, 1 mL FeSO4 solution (9 mM), 1 mL H_2_O_2_ solution (0.3%) and 0.5 mL salicylic acid ethanol solution (9 mM) were mixed with 0.5 mL of sample water solution at various concentrations (0.0625, 0.125, 0.25, 0.5, 1, 2, 4 mg/mL). The mixture was shaken thoroughly and then incubated at 37 °C for 30 min. The absorbance was measured at the wavelength of 510 nm. Vitamin C was used as a positive control. The hydroxyl radical scavenging percentage was calculated by the following equation (Equation (5)):

(5) $$Scavenging\;activity\;(\%)=\frac{A_{0}-\left(A_{a}-A_{b}\right)}{A_{0}}\times 100$$

where *A*_0_ is the absorbance of the blank control (deionized water instead of sample solution), *A_a_* is the absorbance of the sample solution and *A_b_* is the absorbance of the sample under identical conditions as *A_a_* with deionized water instead of FeSO_4_ solution and H_2_O_2_ solution.

#### 4.7.3. ABTS Radical Scavenging Activity

The 2,2′-azino-bis(3-ethylbenzothiazoline-6-sulfonic acid (ABTS) radical scavenging activity of FAPS and APS was determined according to the method of Wang et al. [61] with slight modifications. First, 5 mL of ABTS stock solution (7.4 mM) was added to 88 μL K_2_S_2_O_8_ stock solution (2.6 mM) and allowed to stand for 12–16 h at room temperature in the dark, then ABTS working solution was made by adding deionized water to yield an absorbance of 0.70 ± 0.02 at a wavelength of 734 nm. Next, 200 μL of ABTS working solution was added to 20 μL of the sample at various concentrations (0.0625, 0.125, 0.25, 0.5, 1, 2, 4 mg/mL). The mixture was shaken thoroughly and then incubated at room temperature for 6 min, and then the absorbance was measured at a wavelength of 517 nm. Vitamin C was used as a positive control. The ABTS radical scavenging percentage was calculated by the following equation (Equation (6)):

(6) $$Scavenging\;activity\;(\%)=\frac{A_{0}-\left(A_{a}-A_{b}\right)}{A_{0}}\times 100$$

where *A*_0_ is the absorbance of the blank control (deionized water instead of sample solution), *A_a_* is the absorbance of the sample solution and *A_b_* is the absorbance of the sample under identical conditions as *A_a_* with deionized water instead of ABTS working solution.

#### 4.7.4. Ferric Reducing Power Assay

The reducing power of FAPS was determined according to the method of Zhao et al. [62] with slight modifications as follows: 50 μL of phosphate buffer (pH 6.6) and 50 μL of potassium ferricyanide (1%, *w*/*v*) were added to 50 μL of the sample at various concentrations (0.0625, 0.125, 0.25, 0.5, 1, 2, 4 mg/mL). The mixture was shaken thoroughly and then incubated at 50 °C for 20 min, then 50 μL of trichloroacetic acid (10%, *w*/*v*) and 25 μL of ferric chloride (0.1%, *w*/*v*) were added to the mixtures and the absorbance was measured at a wavelength of 700 nm. The reducing power was calculated by the following equation (Equation (7)):

(7) $$Reducing\;power\;\left(A700\right)=Abs_{1}-Abs_{2}\;$$

where *Abs*_1_ is the absorbance of the sample solution and *Abs*_2_ is the absorbance of the sample under identical conditions as *Abs*_1_ with deionized water instead of ferric chloride solution.

### 4.8. Statistical Analysis

The statistical analysis was conducted using IBM SPSS software (Version 20.0) with one-way analysis of variance (ANOVA). A *p* value < 0.05, denoted by *, was considered statistically significant, while a *p* value < 0.01, denoted by ***, was considered highly significant.

## Figures and Tables

**Table 1:** Central composition design of the three variables in coded units and response values for the extraction yield of crude FAPS.

Run	Extraction Variables			Yield (%)	
	X_1_	X_2_	X_3_	Experimental	Predicted
1	0(60)	1(15)	1(600)	6.82	6.92
2	−1(40)	1(15)	0(480)	6.68	6.66
3	0(60)	0(10)	0(480)	7.45	7.52
4	0(60)	0(10)	0(480)	7.51	7.52
5	0(60)	0(10)	0(480)	7.49	7.52
6	0(60)	0(10)	0(480)	7.58	7.52
7	−1(40)	0(10)	−1(360)	6.55	6.61
8	−1(40)	−1(5)	0(480)	6.81	6.77
9	0(60)	1(15)	−1(360)	6.93	6.89
10	1(80)	0(10)	1(600)	7.12	7.06
11	1(80)	0(10)	−1(360)	6.88	6.95
12	0(60)	−1(5)	1(600)	7.38	7.42
13	0(60)	0(10)	0(480)	7.56	7.52
14	1(80)	1(15)	0(480)	6.81	6.77
15	1(80)	−1(5)	0(480)	7.18	7.20
16	0(60)	−1(5)	−1(360)	7.11	7.01
17	−1(40)	0(10)	1(600)	7.01	6.93

**Table 2:** ANOVA statistics of the quadratic model.

Source	Sum of Squares	df	Mean Square	F-Value	*p*-Value
Model	1.72	9	0.19	24.30	0.0002 ***
X_1_	0.11	1	0.11	14.01	0.0072 ***
X_2_	0.19	1	0.19	24.38	0.0017 ***
X_3_	0.092	1	0.092	11.73	0.0111 *
X_1_X_2_	0.014	1	0.014	1.83	0.2186
X_1_X_3_	0.012	1	0.012	1.53	0.2553
X_2_X_3_	0.036	1	0.036	4.58	0.0696
X_1_^2^	0.70	1	0.70	89.35	<0.0001 ***
X_2_^2^	0.24	1	0.24	30.51	0.0009 ***
X_3_^2^	0.20	1	0.20	25.62	0.0015 ***
Residual	0.055	7	7.883 × 10^−3^		
Lack of Fit	0.044	3	0.015	5.31	0.0703
Pure Error	0.011	4	2.770 × 10^−3^		
Cor Total	1.78	16			
*R*^2^ = 0.9690, *R_adj_*^2^ = 0.9291					

* *p* < 0.05, *** *p* < 0.01.

**Table 3:** The chemical component of FAPS and APS.

Samples	FAPS	APS	*p*
Total carbohydrate (%)	95.38 ± 6.20	90.98 ± 3.80	0.047 *
Proteins (%)	1.26 ± 0.34	6.76 ± 0.87	0.01 *
Sulfate radical (%)	0.98 ± 0.06	1.06 ± 0.04	0.163
Polyphenol (%)	ND	ND	ND
Reducing sugar (%)	4.38 ± 0.08	1.96 ± 0.03	<0.001 ***

Note: ND: Not Detected. * *p* < 0.05, *** *p* < 0.001.

**Table 4:** The molar ratio of monosaccharides of APS and FAPS.

Name	Mean Value of Molar Ratio	
	APS	FAPS
Man	1.06	2.05
GlcN	0.89	0.92
Rib	0.16	0.28
Rha	0.93	0.67
GlcA	0.53	0.23
GalA	1.77	6.78
GalN	0.23	0.09
Glc	336.86	379.72
Gal	16.97	7.75
Xyl	0.52	0.34
Ara	13.90	13.26

**Table 5:** The molecular weight distribution of APS and FAPS.

	Peak Max RT (mins)	Mp (kDa)	Mn (kDa)	Mw (kDa)	PDI (Mw/Mn)
APS	15.650	203.083	126.787	500.539	3.948
FAPS	14.150	1829.540	614.214	1784.347	2.905

## Data Availability

All data are contained within the article.
